# Antibiotic use and the risk of rheumatoid arthritis: a population-based case-control study

**DOI:** 10.1186/s12916-019-1394-6

**Published:** 2019-08-07

**Authors:** Alyshah Abdul Sultan, Christian Mallen, Sara Muller, Samantha Hider, Ian Scott, Toby Helliwell, Lindsay J. Hall

**Affiliations:** 1 Arthritis Research UK Primary Care Centre, Institute for Primary care and Health Sciences, Keele University, Keele, ST5 5BG UK; 2 Haywood Academic Rheumatology Centre, Midlands Partnership Foundation Trust, Staffordshire, ST6 7AG UK; 3 Gut Microbes & Health Programme, Quadram Institute Bioscience, Norwich Research Park, Norwich, NR4 7UQ UK

## Abstract

**Background:**

Antibiotic-induced disturbances of the human microbiota have been implicated in the development of chronic autoimmune conditions. This study aimed to assess whether antibiotic use is associated with the onset of rheumatoid arthritis (RA).

**Methods:**

A nested case-control study was conducted utilising data from the primary care Clinical Practice Research Datalink (CPRD). Patients with an incident diagnosis of RA were identified (1995–2017). Each case was matched on age, gender, and general practice to ≥ 5 controls without RA. Conditional logistic regression was used to examine previous antibiotic prescriptions and RA onset after controlling for confounding factors.

**Results:**

We identified 22,677 cases of RA, matched to 90,013 controls, with a median follow-up of 10 years before RA diagnosis. The odds of developing RA were 60% higher in those exposed to antibiotics than in those not exposed (OR 1.60; 95% CI 1.51–1.68). A dose- or frequency-dependent association was observed between the number of previous antibiotic prescriptions and RA. All classes of antibiotics were associated with higher odds of RA, with bactericidal antibiotics carrying higher risk than bacteriostatic (45% vs. 31%). Those with antibiotic-treated upper respiratory tract (URT) infections were more likely to be RA cases. However, this was not observed for URT infections not treated with antibiotics. Antifungal (OR = 1.27; 95% CI 1.20–1.35) and antiviral (OR = 1.19; 95% CI 1.14–1.24) prescriptions were also associated with increased odds of RA.

**Conclusion:**

Antibiotic prescriptions are associated with a higher risk of RA. This may be due to microbiota disturbances or underlying infections driving risk. Further research is needed to explore these mechanisms.

**Electronic supplementary material:**

The online version of this article (10.1186/s12916-019-1394-6) contains supplementary material, which is available to authorized users.

## Background

Antibiotics are prescribed to treat a wide range of bacterial infections including respiratory, gastrointestinal (GI), and urinary tract infections. In the UK, around 30% of all patients registered in primary care receive at least one antibiotic prescription per year [1]. As well as targeting bacterial pathogens, antibiotics can also disturb the gut microbiota. This highly diverse and dynamic microbial ecosystem plays an integral part in human health, modulating host metabolism and immunity [2–4]. Importantly, the microbiota can be influenced by numerous factors, with antibiotic treatment suggested as among the most significant [5]. Antibiotic treatment can significantly disturb the gut, oral, and skin microbiota, leading to an immediate reduction in microbial abundance and species diversity. There is a significant body of data indicating that antibiotic usage, particularly during childhood, is a major risk factor for increasing susceptibility to infections and development of atopy and inflammatory bowel diseases (ulcerative colitis, Crohn’s disease) [6]. More recently, studies indicate antibiotic usage appears to increase the risk of autoimmune conditions, including type 1 diabetes, autoimmune liver disease, and juvenile idiopathic arthritis (JIA) [7–10].

RA is a chronic autoimmune inflammatory disease, which is characterised by autoimmune antibody production, which directly leads to bone joint destruction, and associated RA pathology. One of the autoantibodies associated with RA (antibodies to citrullinated peptide antigens (ACPA)) is produced in response to bacterial components that closely mimic host cell receptors, e.g. *Porphyromonas gingivalis* and *Aggregatibacter actinomycetemcomitans* [11, 12]. Indeed, prior infections, and gingivitis in particular, have been identified as potential risk factors for RA development [13]. Notably, gingivitis is a very common oral infectious disease in the UK population, with most adults having some form of gum disease [14]. However, the proportion of this population who develop RA is extremely low, suggesting other aetiological factors are at play. Several experimental studies have linked the microbiota to the development of inflammatory arthritis. Germ-free mouse models do not develop inflammatory arthritis, and certain bacteria (e.g. segmented filamentous bacteria, a mouse-specific taxa) have been shown to induce inflammatory T_H_17 responses, which may drive associated immune-mediated pathology and joint destruction [15, 16]. In humans, previous studies have indicated that there are differences in microbiota composition between healthy and disease cohorts. Although RA microbiota profiles reported have varied between different countries [17, 18], all studies have indicated reduced microbial diversity, which may be associated with external factors such as antibiotic usage. Thus, the aim of this study was to investigate the association between antibiotic prescriptions and the onset of RA using a large, UK-based, primary care dataset.

## Methods

### Data source and study population

We used the Clinical Practice Research Datalink (CPRD), a large database containing UK primary care medical records of anonymised patients. This includes coded symptoms, diagnoses, test results, referrals, and prescriptions. CPRD covers more than 7% of the UK population and is a representative of the general UK population in terms of age and sex [19]. Practices contributing data to the CPRD receive training on recording information, and the database is subjected to quality checks. Data are only used when a practice has reached a certain standard of quality. This is defined as being up to standard (UTS). Prescriptions, test results, and referrals are recorded automatically when requested by the treating clinician. It can be expected that all reasons for consultation of any significance are also recorded.

We identified individuals with a first-ever recorded diagnosis of RA between 1990 and 2017. Diagnosis of RA was ascertained by Read Codes, which have been previously shown to be accurate with a high positive predictive value [20, 21]. Each RA case was assigned an index date corresponding to the incident diagnosis date and individually matched to up to five controls on the year of birth, gender, and general practice. Those with less than 5 years of follow-up before the index date were excluded.

### Antibiotic prescriptions

In UK primary care, all prescriptions are recorded automatically when issued by the clinician, including a code for the chapter of the British National Formulary (BNF) under which they were issued. Therefore, all primary care prescriptions appear in the CPRD. All antibiotic prescriptions recorded more than 1 year before the index date were identified using relevant British National Formulary (BNF) chapter headings. We only focused on systemic oral antibiotics, excluding topical antibiotics. Individuals were considered exposed if they received one or more antibiotic prescriptions more than a year before the index date. Antibiotics prescribed in the year before the RA diagnosis were not considered, as they may be prescribed for early RA symptoms.

### Covariates

For cases and controls, we extracted information on body mass index (BMI), alcohol consumption, and smoking status (current or ex-smoker/never smoker) using the latest measure before the index date. BMI was categorised into four categories: underweight (BMI < 18.5), normal (18.5–24.9), overweight (25–29.9), and obese (≥ 30). Charlson index was used to quantify comorbidities before the index date. Finally, we extracted information on commonly occurring infections (upper and lower respiratory tract, urinary tract, GI) before the index date.

Code lists used to define exposures, outcome, and covariates are available from keele.ac.uk/mrr.

### Statistics analysis

Characteristics of the cases and controls were summarised using descriptive statistics (frequencies and percentages). We used conditional logistic regression to assess the association between antibiotic exposure and the risk of incident RA. We adjusted our estimates for a wide range of confounding factors using a multivariable regression model. Missing information on BMI was considered as a separate category and included in the analysis. Recency of antibiotic use was assessed as the time between antibiotic therapy end date and the occurrence of RA. Using similar methods, we assessed the impact of antibiotic type (bacteriostatic/bactericidal; Additional file 1: Table S1) on RA onset.

To explore whether the underlying infection, rather than the antibiotic prescription, is associated with increased risk of RA (confounding by indication), the strength of association between previous infections and RA was assessed and stratified by infection types. Frequency-dependent associations (number of previous infections) with RA were also explored. For this purpose, similar infections recorded less than 30 days apart were considered as a single episode. As upper respiratory tract (URT) infections may or may not warrant antibiotic therapy, we stratified our analysis by treatment modality (antibiotic-treated URT or untreated URT). Treated URT was defined as those prescribed any antibiotics within 30 days of their initial infection code. For each patient, we counted the number of antibiotic-treated and untreated URT infections before the index date and assessed the association with RA. For comparison, we assessed the association between non-bacterial anti-microbial agents (anti-fungal and anti-viral) and the risk of RA.

It may be possible that certain early RA symptoms may have triggered antibiotic therapy (protopathic bias). To account for this bias, we repeated our primary analysis after redefining our index date based on patients’ first referrals to rheumatology clinics within 5 years before their recorded RA diagnosis (similar date was imposed on matched controls). All statistical analysis was conducted using Stata version 14.

### Approvals

This study was approved by the CPRD’s Independent Scientific Advisory Committee (ISAC) reference number: 18_245.

## Results

### Participant characteristics

Twenty-two thousand six hundred seventy-seven cases of incident RA cases were individually matched to 90,013 controls. Baseline characteristics of the study population are summarised in Table 1. Compared to controls, a larger proportion of RA cases were obese (BMI > 30; 27% vs. 32%), were more likely to smoke (18% vs. 21%), and had a higher number of comorbidities (no comorbidities 63% vs. 57%).

**Table 1:** Basic characteristics of cases and controls

Variable	Controls (*n* = 90,013)		RA cases (*n* = 22,677)	
	*N*	%	*N*	%
Year of RA diagnosis				
1995–1999	8086	9	2033	9
2000–2004	15,253	16.9	3849	17
2005–2009	24,713	27.5	6232	27.5
2010–2014	31,699	35.2	7980	35.2
2015–2017	10,262	11.4	2583	11.4
Age at index (years)				
< 40	19,064	21.2	4857	21.4
40–59	20,416	22.7	5095	22.5
60–69	22,257	24.7	5588	24.6
70–79	19,238	21.4	4843	21.4
80–89	8366	9.3	2093	9.2
≥ 90	672	0.7	201	0.9
Mean age (SD)	61.4	(14.1)	61.4	(14.3)
Gender, female	60,620	67.3	15,276	67.4
Median follow-up before RA diagnosis (IQR)	10	(7–14)	10	(7–14)
Body mass index				
Underweight	22,337	24.8	5102	22.5
Normal	2402	2.7	694	3.1
Overweight	28,948	32.2	7456	32.9
Obese	24,610	27.3	7308	32.2
Missing	11,716	13	2117	9.3
Smoking status				
Never/ex-smokers	74,043	82.3	17,840	78.7
Current	15,970	17.7	4837	21.3
Alcohol				
Never/ex-drinker	29,159	32.4	7341	32.4
1–10 units/weeks	46,692	51.9	11,976	52.8
> 10 units/week	14,162	15.7	3360	14.8
Charlson comorbidity index				
0	56,683	63	12,813	56.5
1–2	24,356	27.1	7219	31.8
3–5	7937	8.8	2355	10.4
> 5	1037	1.2	290	1.3

### Antibiotics and RA

RA cases received more antibiotic prescriptions than controls, but over 80% in each group had received a prescription in the 10 years before the index date (Table 2). Penicillins were the most commonly prescribed antibiotics (72% of participants received at least one prescription before index data), followed by macrolides (33%), trimethoprim (31%), cephalosporins (25%), and tetracyclines and quinolones (both 13%). After adjusting for potential confounding factors, the odds of having been exposed to antibiotics were significantly higher in RA cases than in controls (OR 1.60; 95% CI 1.51–1.68). Redefining our index date based on the first referral to the rheumatology did not change our overall finding (adjusted OR = 1.68; 95% CI 1.59–1.76). A frequency-dependent relationship was observed between the number of prescriptions and the odds of RA. The magnitude of association with RA increased with an increasing number of unique antibiotic prescriptions. The timing of antibiotic exposure was important, with higher odds of RA in those last exposed to antibiotics in 1 to 2 years before the index date (OR 1.80; 95% CI 1.70–1.90) than in those last exposed between 5 and 10 years before the index date (OR = 1.57; 95% CI 1.49–1.66).

**Table 2:** Association between antibiotic prescriptions and RA

Variables	Controls		Cases		Crude OR (95% CI)	Adjusted OR^a^ (95% CI)
	*N*	%	*N*	%		
Any antibiotic use						
No prescriptions	16,242	18.0	2369	10.4	Reference	Reference
≥ 1 prescription	73,771	82.0	20,308	89.6	2.02 (1.92–2.12)	1.60 (1.51–1.68)
No. of prescriptions (quartile) (median (IQR))						
1 (1 (1, 2))	20,321	22.6	4128	18.2	1.49 (1.41–1.57)	1.40 (1.32–1.48)
2 (4 (3, 5))	18,923	21.0	4592	20.2	1.85 (1.75–1.96)	1.66 (1.57–1.77)
3 (8 (7, 9))	17,524	19.5	5231	23.1	2.36 (2.23–2.50)	2.04 (1.92–2.17)
4 (19 (14, 29))	17,003	18.9	6357	28.0	3.10 (2.93–3.28)	2.56 (2.39–2.74)
Recency of therapy (therapy termination)^b^						
1–2 years before diagnosis	30,041	35.2	9557	43.9	2.34 (2.23–2.46)	1.80 (1.70–1.90)
2–5 years before diagnosis	26,898	31.5	7016	32.2	1.91 (1.81–2.01)	1.57 (1.49–1.66)
5–10 years before diagnosis	12,183	14.3	2846	13.1	1.70 (1.59–1.80)	1.48 (1.39–1.58)
Antibiotic type						
Bacteriostatic						
Unexposed^c^	43,580	48.4	8778	38.7	Reference	Reference
Exposed	46,433	51.6	13,899	61.3	1.55 (1.50–1.60)	1.31 (1.27–1.36)
Bactericidal						
Unexposed^d^	21,892	24.3	3595	15.9	Reference	Reference
Exposed	68,121	75.7	19,082	84.2	1.80 (1.72–1.87)	1.45 (1.39–1.51)

^a^Adjusted for age, BMI, smoking status, Charlson comorbidity index, alcohol, and infections

^b^Most recent course of therapy

^c^Including those people exposed only to bactericidal antibiotics

^d^Including those people exposed only to bacteriostatic antibiotics

**Fig. 1:**
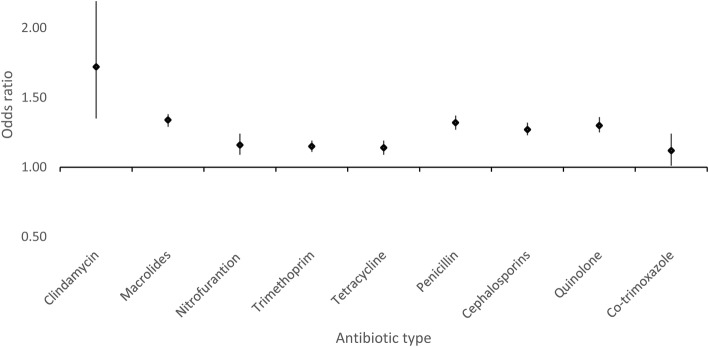
Association between RA and antibiotic class. Comparison of individual antibiotic classes with no antibiotics. Estimates are adjusted for Charlson index, age category, BMI category, smoking, alcohol consumption, and infection types

All classes of antibiotics were associated with higher odds of RA, with clindamycin having the highest odds ratio (Fig. 1). Based on the mode-of-action, the odds of RA were 45% higher in those prescribed antibiotics inducing a bactericidal response (i.e. lyse bacteria) (OR = 1.45; 95% CI 1.39–1.51) compared to those not prescribed antibiotics. The increase in odds was slightly lower with antibiotics inhibiting bacteria (i.e. bacteriostatic) when similar comparisons were made (OR = 1.31; 95% CI 1.27–1.36).

### RA and infection

Cases were more likely to have a record of infection before their RA diagnosis (Table 3). This increased odds of RA among those with a previous history of infection ranged between 7% (urinary tract infection; OR = 1.07; 95% CI 1.02–1.12) and 37% (for LRT infections; OR = 1.37; 95% CI 1.32–1.41). A frequency-dependent association also existed between the number of previous infections and the odds of RA. More than 87% of lower respiratory and urinary tract infections were treated with antibiotics within 30 days. This proportion was lower for URT (74%) and GI infections (21%). Those with antibiotic-treated URT infections were more likely to be RA cases. However, the association was not observed for the number of untreated URT infections.

**Table 3:** Association between the history of infections and RA

Variables	Controls		Cases		OR (95% CI)	Adjusted OR* (95% CI)
	*N*	%	*N*	%		
Infection ever before diagnosis						
Lower respiratory tract	31,400	34.9	10,050	44.3	1.56 (1.51–1.61)	1.37 (1.32–1.41)
Upper respiratory tract	41,786	46.4	12,258	54.1	1.42 (1.38–1.47)	1.26 (1.22–1.31)
Gastrointestinal	8352	9.3	2699	11.9	1.33 (1.27–1.40)	1.19 (1.13–1.25)
Urinary tract	11,037	12.3	3179	14	1.19 (1.13–1.24)	1.07 (1.02–1.12)
Infection episodes						
0	34,899	38.8	6835	30.1	Reference	Reference
1	18,503	20.6	4366	19.3	1.26 (1.21–1.32)	1.22 (1.72–1.28)
2	11,041	12.3	2990	13.2	1.48 (1.41–1.56)	1.42 (1.35–1.49)
3	7091	7.9	2066	9.1	1.63 (1.54–1.73)	1.54 (1.46–1.64)
≥ 4	18,479	20.5	6420	28.3	2.02 (1.93–2.11)	1.86 (1.78–1.94)
No. of treated URT						
0	8466	20.3	2078	17.0	Reference	Reference
1	16,536	39.6	4510	36.8	1.11 (1.05–1.18)	1.10 (1.04–1.17)
2	7052	16.9	2236	18.2	1.29 (1.21–1.38)	1.27 (1.19–1.50)
3	3554	8.5	1233	10.1	1.41 (1.30–1.53)	1.39 (1.28–1.51)
≥ 4	6178	14.8	2201	18.0	1.45 (1.36–1.55)	1.42 (1.32–1.52)
No. of untreated URT						
0	21,981	52.6	6510	53.1	Reference	Reference
1	13,893	33.2	3908	31.9	0.95 (0.91–0.99)	0.95 (0.91–1.00)
2	3714	8.9	1132	9.2	1.03 (0.96–1.11)	1.02 (0.95–1.07)
3	1274	3	385	3.1	1.02 (0.91–1.15)	1.00 (0.89–1.13)
≥ 4	924	2.2	323	2.6	1.18 (1.04–1.34)	1.14 (1.00–1.29)

*Adjusted for age, BMI, smoking status, Charlson comorbidity index, alcohol, and presence/absence of LRT, URT, GII, and UTI

### Additional analysis

A total of 1580 (7%) and 3166 (14%) of RA cases received at least one antiviral and antifungal prescription, respectively, before the index date. After adjusting for confounding factors, including antibiotic use, both antifungal (OR = 1.19; 95% CI 1.14–1.24) and antiviral drugs (OR = 1.27; 95% CI 1.20–1.35) were associated with increased risk of RA.

## Discussion

Using routinely collected primary care data, we have assessed the association between antibiotic use and RA onset. Those exposed to one or more antibiotic prescriptions were 60% more likely to develop RA than their unexposed counterparts. A frequency-dependent association was observed between previous antibiotic prescriptions and RA, and the odds of RA were higher in those with more recent antibiotic exposure. The odds of RA varied by class and mode-of-action of antibiotics. Infection type was also important; those with antibiotic-treated URT infections were more likely to be RA cases. However, the same association was not observed for the number of untreated URT infections. Finally, other anti-microbial agents (i.e. antifungals and antivirals) were associated with increased RA risk.

Respiratory infections showed the strongest association with RA. *Chlamydia pneumoniae*, *Streptococcus pyogenes*, *Streptococcus pneumoniae*, and *Klebsiella pneumoniae* are often associated with respiratory tract infections, and previous studies have linked specific pathogens (e.g. *C. pneumoniae*) with increased levels of circulating autoimmune antibodies that may play a role in RA pathology [22]. However, as numerous pathogens are associated with URT infections, other factors may also play a strong role in disease association. Indeed, our analysis indicates the strongest association is only present in antibiotic-treated cases of URTs (74% patients treated within 30 days of diagnosis), not untreated URT infections, suggesting that associated antibiotic usage is likely to be the main associative factor for the increased incidence of RA in this cohort. A previous case-control study in JIA also suggested that antibiotic-treated URT infections were more strongly associated with JIA than untreated URT infections [8]. It should be noted that antibiotic-treated respiratory infections may be expected to be more severe, thus potentially confounding analysis; nonetheless, the link between the severity of an infection and antibiotic prescription practices is not currently clear. Whilst there is an expectation that this should be the case, numerous previous studies have highlighted that clinician-dependent decisions and preference are the likely driver for an antibiotic prescription for infections that notably are rarely confirmed by microbiological testing (with many cases potentially being of viral origin) [23, 24]. Further analysis of antibiotic usage indicates that all classes increase the risk of RA development, which suggests that provision of antibiotics rather than type/class is associated with RA. The odds ratio for clindamycin was the largest, which was also observed by Arvonen et al. [10] in JIA patients. However, the confidence interval is very wide, which reflects the small numbers of people prescribed with this antibiotic (*n* = 307, 0.27% of the sample); thus, this medication is not expected to have a strong influence on the overall association of antibiotic prescription with RA diagnosis. Breaking down antibiotics into their mode-of-action revealed that there was a stronger association of bactericidals than bacteriostatics with RA (OR 1.45 vs. 1.31), which may correlate with increased gut microbiota disturbances and link to our observation that antibiotic exposure up to 10 years prior to diagnosis was associated with increased risk of RA [5]. Lysis of bacteria by bactericidal antibiotics may also be associated with further microbial compounds circulating systemically and inducing autoimmune antibodies that are associated with RA pathology. However, it is currently unclear if the major driver is infection and/or antibiotic usage; therefore, these observations require extensive additional studies (both case-controlled and mechanistic) to determine direct causation.

The rise in inflammatory, allergic, and autoimmune conditions over the past 20–30 years has been linked to improved diagnostics and a more ‘Western’ lifestyle, which includes the widespread use of antibiotics [9]. Importantly, these antibiotics, whilst crucial in treating serious bacterial pathogens, also affect beneficial microbiota members, leading to a reduction in microbial diversity. In some cases, these disturbances appear to be longer term, whilst in others, the microbiota appears to revert to baseline [25, 26]. However, even short-term changes may interrupt the key immune pathways, which may manifest as symptoms in later-life conditions. Indeed, there is now strong evidence linking antibiotic-induced microbiota disturbances to numerous pathologies. Indeed, IBD is defined as ‘a chronic intestinal inflammation that results from host-microbial interactions in a genetically susceptible individual’, and previous studies have indicated a sevenfold higher risk of IBD development in response to antibiotic use [27]. Furthermore, many mechanistic preclinical studies have provided strong evidence that an antibiotic-disturbed gut microbiota plays a direct role in disease pathology [28]. Similarly, alongside the genetic susceptibility component to RA, it is apparent that additional environmental factors, including microbes, may play a role in disease onset. Horton et al. performed a case-controlled study of antibiotic usage and JIA that indicated that any antibiotic exposure was associated with increased risk of developing JIA [8]. This association was also the frequency of antibiotic use-dependent, supporting our findings that inflammatory arthritis may be associated with antibiotic-related gut microbiota disturbances. Our study is the first to investigate the association between antibiotic usage and RA and suggests that antibiotics may be a major risk factor for RA development.

Small scale studies profiling the microbiota of RA patients have indicated reduced bacterial taxon diversity. A study from the mid-1990s focussing on microbial-derived compounds, i.e. cellular fatty acids, indicated a difference between RA patients and controls, suggesting a microbiota component to RA pathology [29]. Other studies using 16S rRNA microbiota analysis have indicated changes in different bacterial genus ranging from reductions in *Bacteroides*, *Prevotella*, and *Porphyromonas* to another study indicating that a subpopulation of early RA patients had increased prevalence of *Prevotella copri*; however, it should be noted that these observed microbiome changes may also be a result of RA disease state [30, 31]. Using more sensitive microbiota profiling approaches, i.e. shotgun metagenomics (involves sequencing the entire gene content of microbiota samples), Zhang et al. determined that both oral and gut microbial communities are altered in RA patients, with *Bifidobacterium bifidum* and *Haemophilus* spp. reduced and negatively correlated with the levels of serum autoantibodies, whereas *Lactobacillus salivarius* was over-represented, particularly in patients with very active RA [17]. A lung microbiota study, using metataxonomic 16S rRNA profiling, indicated that bronchoalveolar lavage fluid (BAL) from RA patients had reduced species diversity, which correlated with RA-associated lung pathology, and suggests that lung microbiota changes may also be associated with clinical outcomes [32]. However, all these studies were carried out in small numbers of patients (< 80), and none has investigated the association with antibiotic use, thus highlighting the need for further studies in this area.

Alongside bacteria, the microbiota also includes fungi and viruses, and more recent studies have indicated that these microorganisms play a key role in microbial ecosystem structuring and modulating host immune responses [33, 34]. Thus, our findings that increased risk of RA was also observed for patients prescribed other anti-microbial agents (i.e. antifungals, antivirals), albeit a lower risk than antibiotics, suggests that the risk may be in part driven by disturbances in these microbial groups, which warrants further exploration. Additional analysis to assess the potential confounding effect of antifungals and antivirals (data not shown) suggested that prescriptions of the classes of medication were independent of each other.

Our study has a number of limitations. First, there may be misclassification of RA cases, as we were reliant on general practitioners accurately record RA diagnosis. However, previous studies have shown high accuracy of RA recording in primary care; therefore, the risk of misclassification will be minimal [20, 21]. On that note, we considered incident RA cases based on the earliest recording of RA in the patients’ primary care record. This may sometimes not reflect the true date of RA onset as it should be diagnosed by a specialist rheumatologist. Any delay in the recording of RA in primary care data may lead to protopathic bias. However, our finding remained unchanged when we conducted a sensitivity analysis after redefining our index date based on the first referral to a rheumatologist. Second, factors such as parity and breastfeeding are associated with increased risk of RA, which may have confounded our observed association. However, the impact of this confounding will be limited, as the onset of RA is relatively rare in the younger population. Third, we may have underestimated the proportion of individuals exposed to antibiotics, as we did not have information on antibiotics prescribed in secondary care. Given that we found that patients with RA have more comorbidities compared to controls, it is reasonable to assume that RA cases may have had more hospital admissions and subsequently received more secondary care prescriptions. Thus, we may have underestimated the association between antibiotic prescriptions and RA. In addition, there may be confounding by the underlying infection. Whilst increased risk of RA was not observed for those with URT infections not treated with antibiotics, this finding should be interpreted with caution as antibiotics may be a marker of infection severity (albeit with the caveats highlighted above). Finally, we did not determine relative doses of antibiotics taken; therefore, we used frequency of prescriptions to correlate with potential dose and other commonly prescribed drugs, e.g. proton pump inhibitors may be correlated with antibiotic use and in turn confound the observed influence on RA risk (and the microbiota), with additional studies required to explore these complex issues [35]. This finding cannot be generalised to other infections as we have shown that the risk of RA varies by the infection site.

Although we have identified a strong association between antibiotic usage and the onset of RA, there remain many unanswered questions, particularly whether this is related to infections themselves, alterations in the microbiota, or a combination of the two. Further clinical information on the causative pathogens, and their association with the risk of RA, may shed light on this, and further larger microbiota profiling studies, using cutting edge sequencing approaches, may provide a further resolution on the microbes present or absent and links to specific microbial components. However, these studies should also be underpinned by mechanistic investigations that help researchers define what processes are involved, providing an important platform for the development of new prevention or intervention strategies. Indeed, with the identification of microbiota disturbances in several human disease pathologies, modulation of the microbiota has become a target for intervention and treatment. This may also prove to be useful in the context of RA, although 80% of people had a recorded prescription for an antibiotic, the odds of having received an antibiotic were 60% higher in those with RA. To date, there have been a limited number of randomised clinical trials using probiotics (live organisms which, when administered in adequate amounts, confer a health benefit on the host), to treat RA, and outcomes have been inconsistent [36]. However, immune readouts indicate a reduction in inflammatory cytokines (TNF-α and IL-8) and a concurrent increase in IL-10 (an anti-inflammatory cytokine) [37]. We feel this is an important study to highlight other aetiological factors, such as antibiotics, that may contribute to RA risk. Studies of this nature, profiling a huge longitudinal population base, are critical for next stage detailed clinical studies and development of innovative therapies for this debilitating disease, including probing underlying mechanisms and potential associations between microbiota disturbances, infection, and RA onset.

## Additional file

Additional file 1: **Table S1.** Categorisation of antibiotics. (DOCX 13 kb)

## Data Availability

CPRD data were analysed under licence and are not available for public sharing. Access to CPRD is available to all qualified researchers via procedures detailed on the CPRD website (https://www.cprd.com/home/). Applications for data access are submitted to isac@cprd.com.

## Abbreviations

ACPA: Antibodies to citrullinated peptide antigens
BAL: Bronchoalveolar lavage fluid
BMI: Body mass index
BNF: British National Formulary
CI: Confidence interval
CPRD: Clinical Practice Research Datalink
GI: Gastrointestinal
IQR: Interquartile range
ISAC: Independent Scientific Advisory Committee
JIA: Juvenile inflammatory arthritis
LRT: Lower respiratory tract
OR: Odds ratio
RA: Rheumatoid arthritis
SD: Standard deviation
TNF: Tumour necrosis factor
URT: Upper respiratory tract
UTS: Up to standard
