# Supplementary material for: Antibiotic use and the risk of rheumatoid arthritis: a population-based case-control study
Source: BMC Med. 2019 Aug 7;17:154. doi: 10.1186/s12916-019-1394-6 (PMC6685281; doi:10.1186/s12916-019-1394-6)
Supplement: Supplementary file 1 — Table S1. Categorisation of antibiotics. (DOCX 13 kb) [file 12916_2019_1394_MOESM1_ESM.docx]

**Table S1: Categorisation of antibiotics**

| **Bacteriostatic** | **Bactericidal** |
| --- | --- |
| - Clindamycin - Macrolides - Nitrofurantoin - Trimethoprim - Tetracyclines | - Penicillin derivatives - Cephalosporins - Fluoroquinolones - Metronidazole - Co-trimoxazole |
